# Quantification of Reverse Transcriptase Activity by Real-Time PCR as a Fast and Accurate Method for Titration of HIV, Lenti- and Retroviral Vectors

**DOI:** 10.1371/journal.pone.0050859

**Published:** 2012-12-05

**Authors:** Jolien Vermeire, Evelien Naessens, Hanne Vanderstraeten, Alessia Landi, Veronica Iannucci, Anouk Van Nuffel, Tom Taghon, Massimo Pizzato, Bruno Verhasselt

**Affiliations:** 1 Department of Clinical Chemistry, Microbiology, and Immunology, Ghent University, Ghent, Belgium; 2 Centre for Integrative Biology (CIBIO), University of Trento, Trento, Italy; Burnet Institute, Australia

## Abstract

Quantification of retroviruses in cell culture supernatants and other biological preparations is required in a diverse spectrum of laboratories and applications. Methods based on antigen detection, such as p24 for HIV, or on genome detection are virus specific and sometimes suffer from a limited dynamic range of detection. In contrast, measurement of reverse transcriptase (RT) activity is a generic method which can be adapted for higher sensitivity using real-time PCR quantification (qPCR-based product-enhanced RT (PERT) assay). We present an evaluation of a modified SYBR Green I-based PERT assay (SG-PERT), using commercially available reagents such as MS2 RNA and ready-to-use qPCR mixes. This assay has a dynamic range of 7 logs, a sensitivity of 10 nU HIV-1 RT and outperforms p24 ELISA for HIV titer determination by lower inter-run variation, lower cost and higher linear range. The SG-PERT values correlate with transducing and infectious units in HIV-based viral vector and replication-competent HIV-1 preparations respectively. This assay can furthermore quantify Moloney Murine Leukemia Virus-derived vectors and can be performed on different instruments, such as Roche Lightcycler® 480 and Applied Biosystems ABI 7300. We consider this test to be an accurate, fast and relatively cheap method for retroviral quantification that is easily implemented for use in routine and research laboratories.

## Introduction

Retroviral vectors have become an indispensable tool in any modern molecular biology laboratory. They allow stable expression of a gene of interest in dividing cells, as well as stable gene knock-down by expression of short hairpin RNA (shRNA). A subset of vectors, derived from lentiviruses such as human immunodeficiency virus (HIV-) 1 and 2 and feline immunodeficiency virus (FIV), can be used for efficient transduction of non-dividing cells and have therefore received increased attention for both basic research and clinical applications [1], [2], [3], [4]. Although methods for accurate quantification of retroviral vector titers will be indispensable in a clinical setting, also the basic research environment can benefit from a fast and inexpensive method to evaluate the quality of retroviral vector productions. Furthermore, research laboratories investigating the replication of retroviruses, such as HIV, require routine assays to determine retroviral titers after production and during viral infection. Multiple methods for retroviral titer quantification are currently available (see [5], [6] for an overview of lentiviral titration methods), but they often have some inherent drawbacks. Determination of the proportion of transduced/infected cells, by evaluating viral integration or transgene expression, provides a good estimate of the number of functional viral particles, but is time-consuming as it requires transduction/infection of the cells and several days of incubation. Other more rapid methods measure both functional and non-functional viral particles in the supernatant, by quantifying the levels of retroviral Gag protein (such as the HIV p24 protein) and the levels of viral genomic RNA. The former is often done by enzyme-linked immunosorbent assay (ELISA) and consequently has a limited linear range and high cost. The latter relies on quantitative real-time PCR (qPCR)-based amplification of cDNA of virion-associated RNA and requires target-specific primers. Furthermore, both methods are still quite labor-intensive.

An alternative retroviral titration method involves quantification of the reverse transcriptase (RT) activity, which is associated with all retroviral particles. In these assays, an exogenous RNA template is added to the viral supernatant and RT activity is estimated by determining the amount of RNA that is converted to cDNA by the retroviral RT. In the first generation RT assays, cDNA production was monitored by measurement of labeled nucleotide incorporation [7], [8], [9]. Sensitivity was highly increased when a PCR amplification step of the synthesized cDNA was introduced prior to product detection. These types of assays are commonly known as product-enhanced RT (PERT) assays. However, quantification of PCR products still required labor-intensive techniques such as DNA gel electrophoresis, Southern Blot or ELISA [10], [11], [12], [13], [14], [15]. The newest PERT generation therefore uses integrated qPCR techniques for fast cDNA quantification, further increasing both the accuracy and linear range of the assays. Most qPCR based PERT assays use cDNA-specific fluorogenic labeled probes (Taqman® chemistry) for signal generation (F-PERT) [16], [17], [18], [19], [20], although a one-step PERT assay using the more accessible and cost-efficient SYBR Green-I chemistry (SG-PERT) was also recently developed [21]. PERT assays are now routinely used for detection of retroviral contaminants in biological products intended for human use [22], [23], [24], [25], [26], [27], [28], [29], [30], [31]. However, in basic research environment, the implementation of real-time PCR based PERT assays is still limited, despite their low cost and fast procedure.

In this paper, we present an adapted version of the SG-PERT assay described before by Pizzato et al. [21]. The assay was adapted for use with different commercial ready-to-use SYBR Green I qPCR reaction mixes, to allow an easy implementation of the assay in any research lab with qPCR experience and to avoid possible compositional variation inherent to in house prepared qPCR mixes. In addition, RNA from bacteriophage MS2, which also lacks a DNA phase in its life-cycle, was used instead of RNA from the Brome Mosaic Virus (BMV) that was used as a template in the original assay, but is no longer commercially available. Sensitivity and specificity of the assay were determined, as well as the variation on repeated RT activity measurement within and between runs. We used the assay to evaluate the informative value of the RT activity for lentiviral titer determination, by comparing it to more commonly used titration methods. We observed excellent correlation with the p24 antigen concentration in both replication-competent HIV-1 virus supernatant and replication-incompetent HIV-based lentiviral vector preparations, as well as with the levels of transducing units or infectious units. This particular assay outperformed p24 ELISA by its lower inter-run variation, lower cost and higher linear range. Furthermore, it was far less time-consuming than both p24 ELISA and determination of transducing or infectious units. We therefore believe that this assay forms an attractive alternative to routine retroviral and lentiviral titer determination in routine and research laboratories.

## Materials and Methods

### Cell Culture

Human lymphoblastoid Jurkat E6.1 (ATCC Cell Biology Collection, Manassas, VA, USA), Jurkat CD4 CCR5 (Programme EVA Centre for AIDS Reagents, NIBSC, UK), human embryonal kidney 293T (DZSM, Braunschweig, Germany), 293TN (System Biosciences, Mountain View, CA, USA), Phoenix-Amphotropic packaging (Phoenix A) cells (Dr P. Achacoso and Dr G.P. Nolan, Stanford University School of Medicine, Stanford, CA, USA) [32] and P4.R5 MAGI cells (AIDS Research and Reference Reagent Program, Division of AIDS, NIAID, NIH, Germantown, MD, USA) [33] were cultured at 37°C in a 7% CO2 humidified atmosphere, in IMDM complete medium: Iscove’s modified Dulbecco’s medium (Life Technologies, Merelbeke, Belgium) supplemented with 10% fetal bovine serum (Hyclone, Thermofisher Scientific, Waltham, MA, USA), 100 U/mL penicillin (Life Technologies) and 100 µg/mL streptomycin (Life Technologies).

### Production of Replication-competent HIV-1 Virus

Replication-competent HIV-1 virus was produced by transfection of 293T cells with one of the following pNL4-3 proviral constructs: the NLENG1-IRES vector (kindly provided by Dr. D.N. Levy, New York University college of Dentistry, New York, NY) [34], the NL4-3-IRES-HSA vector (kindly provided by Dr. M.J. Tremblay, Faculté de Médecine, Université Laval, Québec, Canada) [35] or the HIV-1 NL4-3-IRES-eGFP vector (kindly provided by Dr. F. Kirchhoff, Institute of Virology, University of Ulm, Ulm, Germany) [36]. Transfection was performed with Calcium Phosphate Transfection Kit (Life Technologies) or JetPei® (Polyplus, Sélestat, France), according to manufacturer’s instructions. Viral supernatant was harvested 48 hours or 72 hours after transfection and centrifugated at 900 g for 10 min, to clarify the supernatant from remaining cells. High-titer viral supernatant, that was used to produce a standard curve for the SG-PERT assay, was obtained by infection of Jurkat CD4 CCR5 cells with HIV-1 (140 ng p24 equivalent per mL) and subsequent collection of the culture medium 12 days after infection. During infection, culture medium was refreshed every two or three days.

### Production of Replication-incompetent Lentiviral and Retroviral Vectors

All replication-incompetent lentiviral vectors used in this study were produced using the pLKO.1-puro Non-Mammalian shRNA control plasmid from the Sigma MISSION® product line (Sigma-Aldrich, Bornem, Belgium), in which the puromycin resistance gene was replaced by an eGFP encoding sequence. 293T or 293TN cells were transfected with this plasmid using Calcium Phosphate Transfection Kit, Lipofectamine® 2000 (Life Technologies) or Fugene® 6 (Promega, Leiden, The Netherlands). HIV packaging genes and the VSV-G heterologous viral envelope gene were provided in the cells by simultaneous cotransfection with either the MISSION® Lentiviral Packaging Mix (Sigma-Aldrich) or the p8.91/pMD.G plasmids [32] and virus was harvested two days after transfection. The Moloney Murine Leukemia Virus (MoMLV)-based retroviral vector was produced by transfection of Phoenix Amphotropic packaging cells with the LZRS-IRES-EGFP vector using the Calcium Phosphate Transfection kit, as described before [37], [38].

### Reagents Required for the SG-PERT Assay

MS2 RNA was purchased from Roche Diagnostics (Vilvoorde, Belgium; Catalog #10165948001). Primers to amplify MS2 cDNA in the SG-PERT reaction were obtained from Eurogentec (Seraing, Belgium) and had the following sequence: FWD (5′-TCCTGCTCAACTTCCTGTCGAG-3′) and REV (5′-CACAGGTCAAACCTCCTAGGAATG-3′), as published [19]. Ribolock™ RNase inhibitor was from Fermentas (St. Leon-Rot, Germany; Catalog # EO0381). For SG-PERT on the LightCycler® 480 (Roche Diagnostics), the LightCycler® 480 SYBR Green I Master mix from Roche was used as reaction mix (Catalog #04707516001). For SG-PERT on the ABI 7300 real-time PCR system (Applied Biosystems, Foster City, CA, USA), a reaction mix was made using the ROX-containing qPCR Core kit for SYBR Green I from Eurogentec (Catalog #RT-QP73-05). Recombinant HIV Reverse Transcriptase was purchased from Ambion (Life Technologies, Catalog # AM2045). 2× concentrated lysis buffer was composed of 0.25% Triton X-100, 50 mM KCL, 100 mM TrisHCL pH 7.4, 40% glycerol and prepared as described previously [21]. 2 µL of RNAse inhibitor was added per 100 µL of the 2× lysis buffer immediately prior to use.

### SG-PERT Assay

Cell-free viral supernatant was generally used without prior dilution as input for the assay. 10-fold dilution series of viral supernatant or HIV recombinant RT were generated in IMDM complete. 5 µL of the viral supernatant or recombinant RT solution was added to a well of a 96-well U-bottom plate (Beckton Dickinson, Erembodegem, Belgium) and mixed with 5 µL of 2× concentrated lysis buffer, already containing RNAse inhibitor. Samples were incubated for 10 minutes at room temperature and subsequently diluted by addition of 90 µL nuclease-free water (Life Technologies). After brief centrifugation, the lysates were resuspended and used as input for the assay. For SG-PERT assays on the LightCycler® 480 instrument, 9.6 µL of the lysate was transferred to a 384-well plate (LightCycler® 480 Multiwell Plates 384, white, Roche Diagnostics), that already contained 10.4 µL of a reaction mix consisting of 10 µL 2× Roche SYBR Green I Master mix, 0.1 µL 10× diluted RNAse inhibitor, 0.1 µL MS2 RNA and 0.1 µl of both the MS2 FWD and REV primer (100 µM, to obtain final concentration of 500 nM in 20 µL reaction volume). After brief centrifugation of the plate, the reaction was carried out according to the following program: 20 minutes (min) at 42°C for RT reaction, 5 min at 95°C for activation of FastStart Taq DNA polymerase and 40 or 50 cycles of amplification: 5 seconds (sec) at 95°C for denaturation, 5 sec at 60°C for annealing and acquisition, 15 sec at 72°C for elongation. Fluorescence acquisition was done at the end of annealing phase in our experiments, but can alternatively be done at the end of elongation phase. For SG-PERT assays on the ABI 7300 qPCR system, 9 µL of the lysate was added to a 96-well plate (MicroAmp Optical 96-well reaction plate, Applied Bisosystems) together with 11 µL of a reaction mix consisting of 10.6 µL mastermix from the Eurogentec qPCR core kit for SYBR Green I (2 µL 10× reaction buffer, 1.4 µL of 50 mM MgCl2, 0.8 µL of 5 mM dNTP mix, 0.1 µL of HotGoldSTar Taq polymerase, 0.6 µL SYBR Green I and 5.7 µL of nuclease-free water.), 0.1 µL 10× diluted RNAse inhibitor, 0.1 µL MS2 RNA and 0.1 µl of both the MS2 FWD and REV primer (100 µM). Following reaction conditions were used on the ABI 7300 instrument: 20 min RT reaction at 42°C, 2 min activation of the HotGoldStar Taq enzyme at 95°C and 40 cycles of amplification: 5 sec denaturation at 95°C, 30 sec annealing and acquisition at 60°C, 15 sec elongation at 72°C.

All reagents were kept on ice or on a cooling block during preparation of the assay. For each sample lysate, an SG-PERT reaction was always performed in duplo. Cycles of quantification (Cq) values were generated by the software of the qPCR instruments, after manual threshold determination for the ABI 7300 instrument and according to the second-derivative maximum method for the LightCycler® 480. Melting peaks were calculated automatically by the software of both instruments.

To perform absolute quantification of RT activity values, a standard curve of replication-competent HIV-1 containing supernatant with known RT activity levels was run in parallel in each assay and values were extrapolated from the obtained Cq values. The standard curve was produced by serial dilution of a large batch of high-titer supernatant. Dilutions were aliquoted for use in different SG-PERT assays, to avoid loss of RT activity by repeated freeze-thaw cycles. RT activity values of the standard curve were determined by running a dilution series of commercial recombinant RT in parallel in at least four independent experiments.

### Calculations and Statistics

Standard deviation (STDEV), coefficient of determination (R^2^) and regression equations were calculated with Excel 2007 (Microsoft). Coefficient of variation (CV) was calculated as follows: (STDEV/AVERAGE) x 100%. Column scatter plots were created with GraphPad Prism version 5.04. Statistical significance of difference between inter-run coefficient of variation of p24 ELISA test and SG-PERT assay was analyzed with the one-tailed non-parametric Mann-Whitney U test (GraphPad Prism).

### Determination of p24 Concentration, Transducing and Infectious Units in Viral Supernatant

Concentration of the p24 antigen was measured in HIV-1 or HIV-based lentiviral vector containing supernatant with the INNOTEST® HIV Antigen mAb ELISA kit (Innogenetics, Zwijnaarde, Belgium), according to manufacturer’s instructions. Multiple dilutions of each sample were tested, to ensure that concentration was within the linear range of the assay. For correlation analysis of p24 antigen and RT activity values, all samples were measured within the same ELISA assay, to avoid the introduction of inter-run variation. For lentiviral vectors, the number of transducing units per volume of supernatant (TU/mL) was determined by transduction of Jurkat E6.1 cells with a limiting dilution series of each sample, using polybrene (8 µg/mL; Sigma-Aldrich) and spinoculation (30 min, 950 g, 32°C). The percentage of transduced cells was determined 72 hours after transduction by FACS analysis (MACSquant® Analyzer, Miltenyi Biotec, Leiden, The Netherlands) of eGFP expression. Vector titers (TU/mL) were calculated in cultures with 0.5% to 4% of eGFP expressing cells according to following formula: { (% eGFP expressing cells/100) x number of cells at moment of transduction x dilution factor }/volume of viral supernatant used for transduction (mL).

For replication competent HIV-1 virus, relative levels of infectious units (IU) were determined by single-cycle infection of P4.R5 MAGI indicator cells. Briefly, 10,000 cells were plated per well of a 96-well flat bottom plate (Beckton Dickinson). Twenty-four hours after plating, HIV-1 NL4-3 viral supernatant was added to the cells in the presence of 1 µM of the HIV protease inhibitor ritonavir (AIDS Research and Reference Reagent Program, Division of AIDS, NIAID, NIH, Germantown, MD, USA), to avoid multiple rounds of infection. Cells were subsequently spinoculated at 950 g for 90 min at 32°C. Forty-eight hours after infection β-galactosidase activity was assessed using a colorimetric assay (Mammalian β-galactosidase Assay kit; Thermofisher Scientific) according to manufacturer’s instructions. Optical density at 405 nm was quantified using a Versa Max Plate Reader (Molecular Devices, Sunnyvale, CA, USA) and obtained values were corrected for background signal by subtraction of the optical density value obtained with non-infected cells. For correlation analysis of RT activity and relative levels of infectious units, we used cell-free HIV NL4-3 viral supernatant harvested 48 hours or 72 hours after transfection, with the Calcium Phosphate Transfection Kit, of 293T with either the NLENG1-IRES vector or the NL4-3-IRES-HSA vector. To ensure that β-galactosidase activity levels were within the linear range of the assay, the viral concentration in the culture was limited to maximum 360 mU RT activity/mL. To determine absolute levels of infectious units/mL, P4.R5 MAGI cells were infected with serial dilutions of HSA encoding HIV-1 virus in presence of ritonavir. Forty-eight hours after single-cycle infection, cells were stained with APC (allophycocyanin)-labeled anti-mouse-CD24 antibody (HSA, heat stable antigen; clone M1/69, BioLegend, San Diego, CA, USA) and the percentage of infected cells was determined by FACS analysis (FACSCalibur flow cytometer; Becton Dickinson)of HSA expression. Viral titers (IU/mL) were calculated according to the following formula: {(% HSA expressing cells/100)×number of plated cells × dilution factor}/volume of viral supernatant used for infection (mL).

## Results

### Sensitivity and Specificity of the SG-PERT Assay

**Figure 1:**
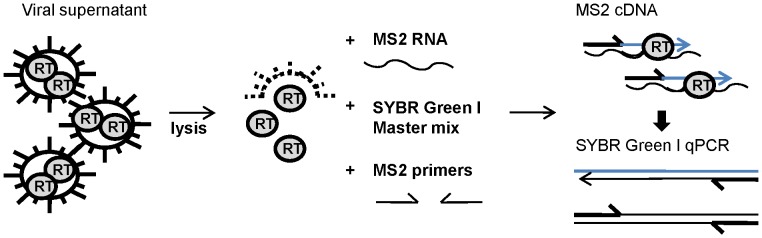
Principle of the SG-PERT assay. Cell-free retrovirus containing supernatant is lysed and added to a reaction mix containing the MS2 RNA template, MS2 complementary primers and a SYBR Green I qPCR mastermix. During a one-step reaction, the reverse transcriptase (RT) enzymes derived from the retroviral particles will convert the MS2 RNA into cDNA and cDNA is subsequently quantified by qPCR amplification of the MS2 cDNA. The amount of synthesized cDNA represents the level of RT activity in the viral supernatant and is thereby a measure of the amount of retroviral particles.

**Figure 2:**
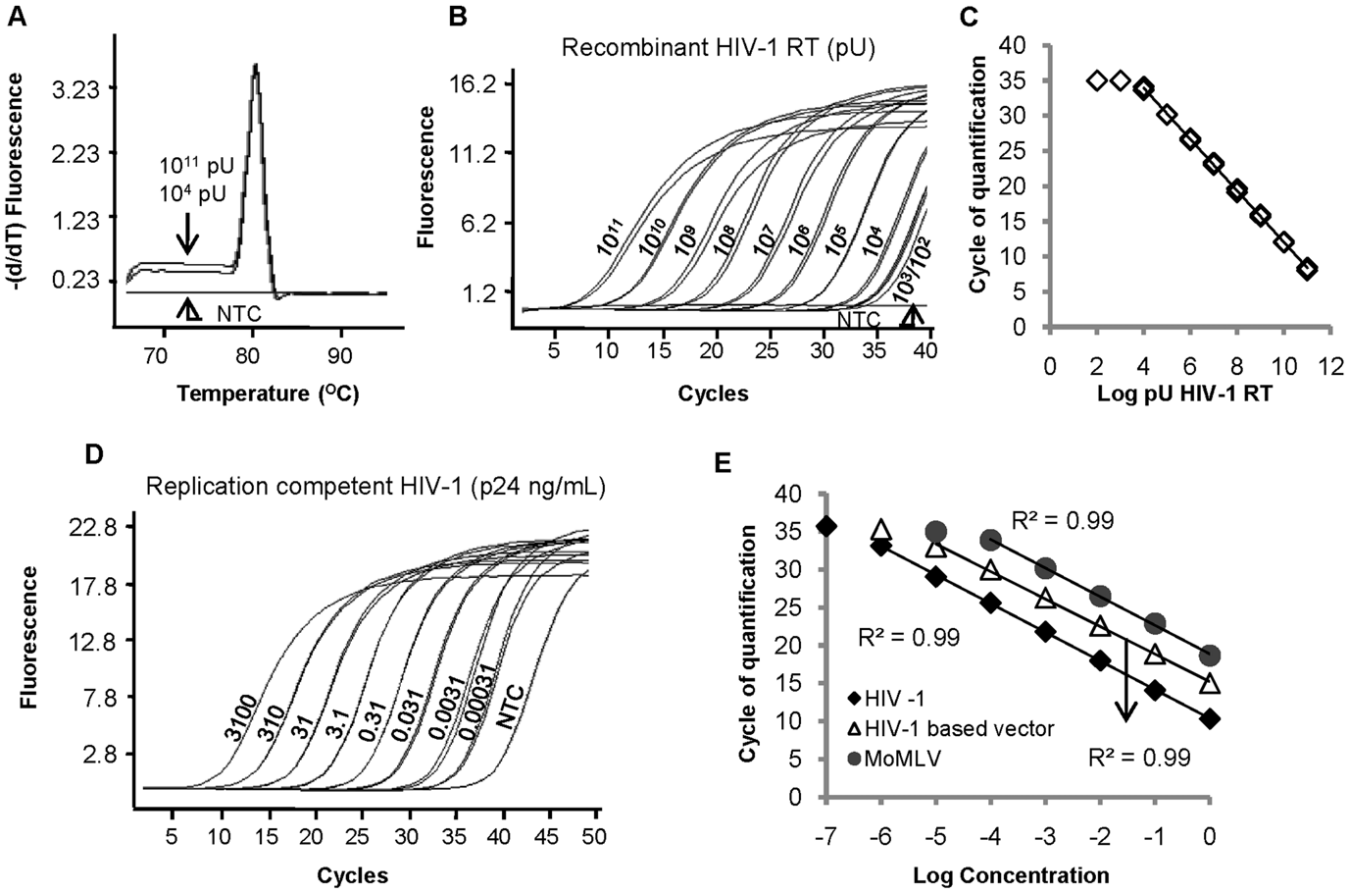
Sensitivity and specificity of the SG-PERT assay. (A) Melting curves of PCR products obtained by SG-PERT assay on the LightCycler® 480 when using 10^11^ or 10^4^ pU recombinant HIV-1 RT or nuclease-free water (non-template control = *NTC*) as input for the assay, as indicated. (B, D) Amplification curves of indicated amount of (B) recombinant HIV-1 RT (pU), (D) replication competent HIV-1 (NL4-3 strain) (ng p24/mL) or nuclease-free water (*NTC*) obtained by SG-PERT on the LightCycler® 480. (C,E) Relation between input of (C) recombinant HIV-1 RT, (E) replication competent HIV-1 (*HIV-1*), HIV-1 based lentiviral vectors (*HIV-1 based vector*) or Moloney Murine Leukemia-based retroviral vectors (*MoMLV*) and obtained cycle of quantification (Cq) values by SG-PERT on the LightCycler® 480. Viral titers in the undiluted samples in (E) (value of “0” on x-axis) were 3,100 ng p24/mL for the replication competent HIV-1 virus, 1.12×10^7^ transducing units/mL (TU/mL) for the HIV-1 based viral vector and 4.9×10^5^ TU/mL for the MoMLV-based vector. Only input levels within linear range of the assay were included for correlation analysis.

A one step SYBR Green I-based real-time PERT assay (SG-PERT) was developed, that uses MS2 RNA as a substrate for reverse transcription and commercially available ready-to-use reaction mixes for MS2 cDNA quantification by SYBR Green I-based qPCR (Figure 1). We first performed experiments using the Roche Master Mix for qPCR on the LightCycler® 480. Inherent to such a SYBR Green I-based detection system, is the possible contribution of non-specific PCR fragments to the measured signal. To evaluate the presence of any non-specific products, we performed a melting curve analysis on the PCR products. Only a single PCR fragment with a melting peak of 80.6°C was detected in the final PCR product of both highly and weakly positive samples (Figure 2A).

In order to determine the sensitivity and linear range of the assay, 10-fold serial dilutions of a recombinant HIV-1 reverse transcriptase (RT) enzyme were used as input for SG-PERT. Amplification of the MS2 substrate correlated with the input amount of recombinant RT (Figure 2B). When plotting the RT input against the obtained Cq, the correlation was linear over 7 orders of magnitude, ranging from 10^11^ pU to 10^4^ pU recombinant HIV-1 RT per reaction (Figure 2C). Lower amounts of recombinant RT (10^3^ and 10^2^ pU) were still detected, but Cq values were considered to be outside the linear range of the assay. Furthermore, when using nuclease-free water (NFW) as input for the assay, a weak signal was occasionally obtained after 37 or more cycles (Figure 2D). This signal might indicate a weak background reverse transcriptase activity of the Taq DNA polymerase in the Roche mix or might be caused by carry-over contamination between SG-PERT experiments. Since Cq values obtained in reactions with 10^3^ and 10^2^ pU input HIV-1 RT enzyme were not consistently above these occasional background values, the detection limit of this assay is considered to be 10^4^ pU recombinant HIV-1 RT.

Subsequently, the ability of the assay to measure the RT activity associated with complete viral particles was tested. When using cell-free supernatant of replication competent HIV-1 infected Jurkat CD4 CCR5 cells as input for the assay, a linear correlation between the input virus dilution and Cq values was again obtained over six 10-fold serial dilutions of the original supernatant. Similar to the results with recombinant HIV-1 RT, higher dilutions of viral supernatant were still detectable (Cq ≥35), but outside the linear range of the assay (Figure 2D and 2E). The undiluted sample used here, had a p24 antigen concentration of 3,100 ng/mL according to ELISA measurement. Therefore, this SG-PERT assay can detect and quantify RT activity in HIV-1 supernatant with a p24 equivalent as low as 0.0031 ng/mL. Since only 0.48 µL of supernatant is used per reaction, this corresponds to a detection limit of 1.5 fg p24 or ±20 viral particles (assuming that an HIV core is composed of 2,000 p24 capsid molecules [6]). Alternatively, when the lowest detectable number of virions is calculated based on virion associated RT activity (400- 200 pU/virion [9], [18]), a similar detection limit of 25–50 virions is obtained.

Since RT activity is associated with all retroviruses, a more prominent application of this assay can be the quantification of recombinant lenti- and retroviral vectors, for instance to check the quality of retroviral production. We used two types of commonly used replication-defective viral vectors, HIV-based and MoMLV-based, to evaluate the correlation between vector concentration and RT activity measured by SG-PERT. Both the RT activity associated with HIV-based MISSION® lentiviral particles as well as the activity of the MoMLV RT enzyme could be detected in supernatant of lentivirus producing 293T cells and retrovirus-producing Phoenix A packaging cells respectively. Activity of both enzymes showed a linear correlation with the input vector dilution over four to five 10-fold dilutions (Figure 2E). When plotting the input concentration versus the obtained Cq values, a curve with similar slope was obtained for recombinant RT, replication competent HIV-1, HIV-based lentiviral vectors and MoMLV-based retroviral vectors (Figure 2C and Figure 2D; varying from −3.6 to −3.8). Therefore, PCR efficiencies must be in the same range and the linear range for retro- and lentiviral vector quantification might be similar to the one of recombinant RT quantification. In order to express the sensitivity of viral vector quantification by the SG-PERT assay, the functional titer of both the lenti- and retroviral vector supernatant used here was determined by limiting dilution titration on Jurkat E6-1 cells and was found to be resp. 1.12×10^7^ transducing units (TU)/mL and 4.9×10^5^ TU/mL. Since the assay could quantify a 10^5^ dilution of the HIV-based lentiviral supernatant, a sensitivity of 1.12×10^2^ TU/mL or 0.056 TU/reaction can be assumed. Similarly, for MoMLV-based retroviral vector supernatant, quantification of up to 49 TU/mL or 0.024 TU/reaction is possible.

### Inter- and Intra-run Variation of the SG-PERT Assay

When determining RT activity in lenti- or retroviral supernatant with the SG-PERT assay, absolute quantification can be done by running a standard curve of recombinant reverse transcriptase in the same assay as the sample of interest. For frequent use of the assay, a cheaper alternative is the use of a retroviral standard curve. In this case, the RT activity of high titer retroviral supernatant is determined once, using a standard curve of recombinant reverse transcriptase. For subsequent assays, a dilution series of this retroviral supernatant can be used as standard curve and absolute quantification can be done by using the known RT activity of the retroviral standard curve.

**Figure 3:**
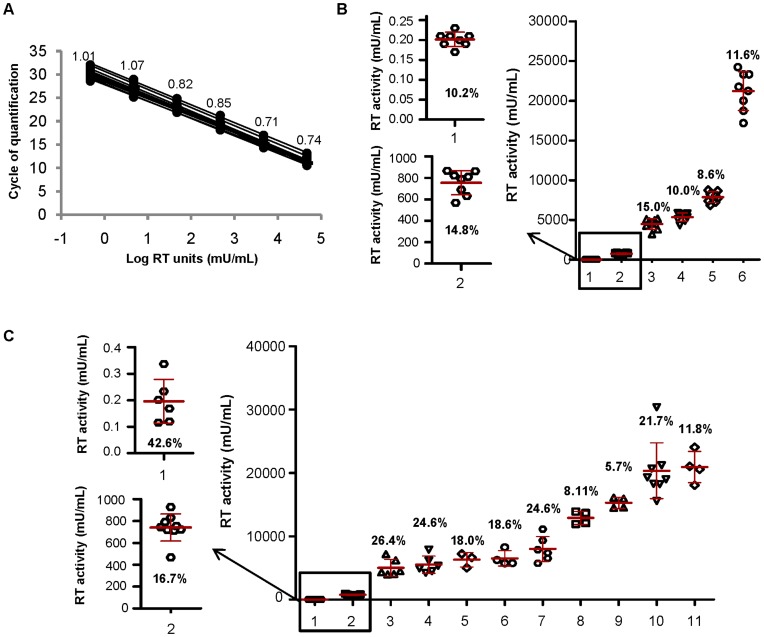
Intra- and inter-run variation of the SG-PERT assay. (A) Standard curve composed of a pre-made six 10-fold serial dilution series of replication-competent HIV-1 containing supernatant measured in 12 independent SG-PERT experiments. For each experiment obtained Cq values are plotted versus the RT activity in each sample. RT activity values were determined by running a dilution series of recombinant HIV-1 RT in parallel. Standard deviation on the obtained crossing point values is indicated for each dilution. (B) RT activity values obtained for 8 repeated measurements of different HIV-1 samples (sample number 1 to 6) within the same run. The average RT activity value for each sample is indicated by a red line, error bars represent standard deviation on the obtained RT activity values. Numbers indicate intra-run variation for each sample, expressed as percentage of the average RT activity values (coefficient of variation). (C) RT activity values obtained for different HIV-1 samples (sample number 1 to 11) in at least 3 independent SG-PERT experiments. The average RT activity value for each sample is indicated by a red line, error bars represent standard deviation on the obtained RT activity values. Numbers indicate inter-run variation for each sample, expressed as percentage of the average RT activity values (coefficient of variation). Experiments were performed on the LightCycler® 480.

To obtain such a standard curve, supernatant of HIV-1 infected Jurkat CD4 CCR5 cells with an RT activity of 47,000 mU/mL was serially diluted and aliquots of each dilution were stored for use in different assays. Cq values of this standard curve, obtained in 12 independent SG-PERT experiments on the LightCycler® 480, are shown in Figure 3A. They were found to be highly reproducible, with a standard deviation of maximum 1 Cq value over the different assays for each dilution point, corresponding to a variation of two-fold over the measurement average. If a precise determination of RT activity is not required, it is therefore possible to only occasionally include a standard curve in the SG-PERT experiment and directly derive the RT activity from the obtained Cq value instead (using a fixed standard curve for calculations).

Subsequently, the reproducibility of RT activity quantification using the SG-PERT assay and the HIV-1 standard curve, was evaluated on the LightCycler. To determine intra-run reproducibility, 8 aliquots from each of 6 different HIV-containing samples were separately lysed and RT activity in the different lysates was determined in the same run. The coefficient of variation (CV) ranged between 8.6% to 15% of the RT activity (Figure 3B), with an average CV of 12%. Inter-run variation was evaluated on 11 different HIV-1 containing samples by determining their RT activity in at least 3 independent SG-PERT experiments. We found an average CV of 19.9%. Except for the sample with the lowest titer, CV was lower than 30%. The sample with very low RT activity (0.2 mU/mL; sample 1 in Figure 3C) showed a CV of 43%, which might indicate a decreased reproducibility of the SG-PERT measurement in the region close to the detection limit of the assay (Figure 3C).

### Comparison of the SG-PERT Assay with Other Lentiviral Titration Methods

Currently, the most frequently used methods for lentiviral titer determination measure the concentration of the p24 antigen in the supernatant or determine the number of transducing units (TU) or infectious units (IU) per volume of the supernatant. The latter can be done by assessing the level of transduced or infected cells after limiting dilution of replication incompetent or replication competent lentiviruses respectively. Alternatively, for HIV viruses, relative levels of infectious units are often determined by single-cycle infection of indicator cell lines. To evaluate the informative value of the RT activity determined by SG-PERT, we investigated its correlation with the p24 antigen concentration levels and the levels of TU or IU in the same samples.

**Figure 4:**
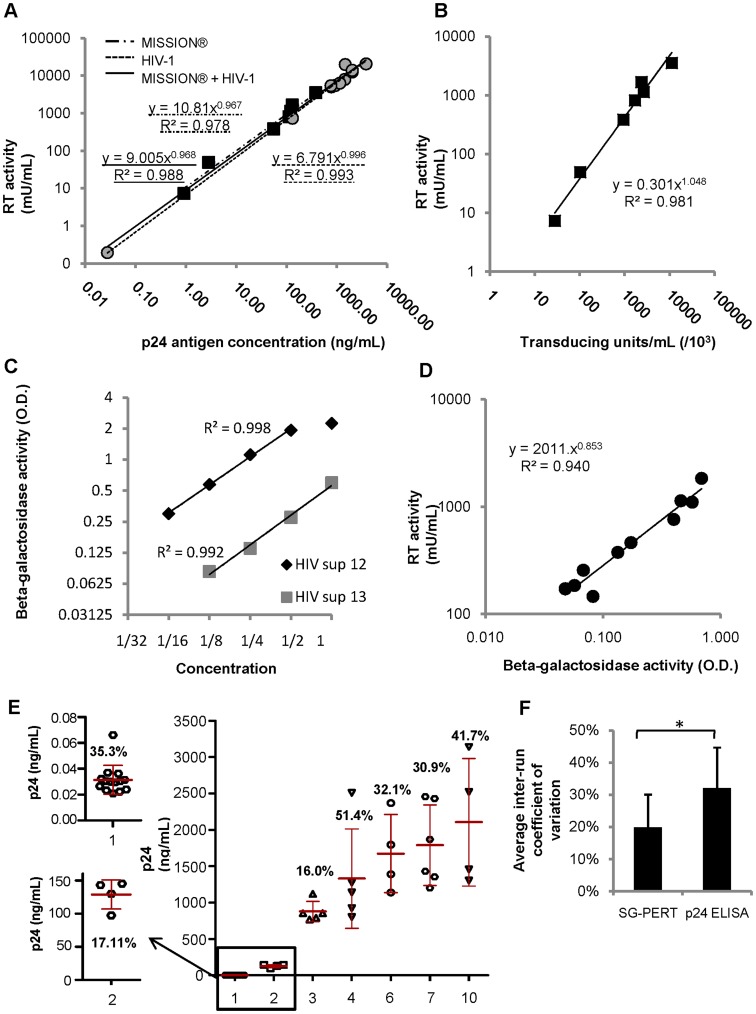
Comparison of SG-PERT assay with other lentiviral titration methods. (A) Correlation between RT activity levels measured with SG-PERT and p24 antigen concentration levels measured with ELISA in supernatant containing replication-competent HIV-1 virus or HIV-1 based MISSION lentiviral vectors. Coefficient of determination and correlation functions are shown for HIV-1 virus (- - -) and lentiviral vectors (-·-·) separately or combined (-). To avoid introduction of interrun variation, p24 ELISA measurements for all samples were performed in the same experiment. (B) Correlation between RT activity levels measured by SG-PERT and levels of transducing units determined by limiting dilution on Jurkat E6.1 cells in HIV-1 based MISSION® lentiviral vector preparations. (C) Relation between the level of β-galactosidase activity, as a marker of HIV-1 Tat production and the relative amount (serial dilution) of replication competent HIV-1 NL4-3 virus used to infect the cells, in the culture of P4.R5 MAGI indicator cells 48 h after single-cycle infection. Viral input in the undiluted samples (value of “1” on x-axis) was 1.76×10^3^ mU RT/mL for HIV sup 12 and 2.33×10^2^ mU RT/mL for HIV sup 13. For HIV sup 12, the undiluted sample was outside the linear range of the assay and was therefore not included for calculation of the determination coefficient (R^2^) shown in panel C. (D) Correlation between the level of β-galactosidase activity in the culture of P4.R5 MAGI indicator cells 48 h after single-cycle infection with different productions of HIV-1 NL4-3 supernatant and the RT activity measured in the supernatant by SG-PERT. (E) p24 antigen concentration values obtained for different HIV-1 containing samples in at least 3 independent ELISA experiments. Sample numbers in x-axis correspond to those in Figure 3C. The average p24 concentration value for each sample is indicated by a red line, error bars represent standard deviation on the obtained p24 concentration values. Numbers indicate inter-run variation for each sample, expressed as percentage of the average p24 concentration (coefficient of variation = CV). (F) Average inter-run coefficient of variation for SG-PERT and p24 ELISA assays, calculated from the CV’s obtained in Figure 3C and Figure 4E respectively. Error bars indicate standard deviations. The asterix (*) indicates a statistical significant difference between the two values according to a one-tailed Mann-Withney U test (p-value = 0.0426).

Supernatants with HIV-1 virus (NL4-3 strain) or replication incompetent HIV-based MISSION® lentiviral particles were used for both p24 antigen concentration and RT activity determination by ELISA and SG-PERT respectively. We found a very strong correlation between the obtained p24 and RT values, both for combined and separate analysis of the lentiviral vectors and HIV-1 viruses. The two values correlated with each other according to a power function, with a power number very close to 1, indicating an almost linear relation. The ratio between the RT and p24 values was calculated, that seemed higher for the lentiviral vectors compared to the replication competent HIV-1 viruses (Figure 4A and Table 1). Of note, the excellent correlation between the p24-RT values of HIV-1 viral supernatants was not caused by the sample with very low titer, since the coefficient of determination (R^2^) was still 0.92 if this sample was removed from the analysis (data not shown).

**Table 1:** Evaluation of different lentiviral titration methods.

	RT activity(mU/mL)	p24 (ng/mL)	ratio RT/p24	TU/mL	p24 based# VP/mL	RT based# VP/mL	ratio p24 based/RT based # VP	ratio TU/p24based # VP	ratio TU/RTbased # VP
HIV-1 sup 1	1.96E-01	2.78E-02	7.0		3.33E+05	6.52E+05	1.95		
HIV-1 sup 2	7.41E+02	1.30E+02	5.7		1.56E+09	2.47E+09	1.58		
HIV-1 sup 3	5.03E+03	7.69E+02	6.5		9.23E+09	1.68E+10	1.82		
HIV-1 sup 4	5.44E+03	7.57E+02	7.2		9.08E+09	1.81E+10	2.00		
HIV-1 sup 5	5.52E+03	9.26E+02	6.0		1.11E+10	1.84E+10	1.66		
HIV-1 sup 6	6.34E+03	1.14E+03	5.6		1.37E+10	2.11E+10	1.55		
HIV-1 sup 7	8.00E+03	1.43E+03	5.6		1.72E+10	2.67E+10	1.55		
HIV-1 sup 8	1.26E+04	2.00E+03	6.3		2.40E+10	4.19E+10	1.75		
HIV-1 sup 9	1.39E+04	2.01E+03	6.9		2.41E+10	4.64E+10	1.92		
HIV-1 sup 10	2.00E+04	1.46E+03	13.7		1.75E+10	6.65E+10	3.81		
HIV-1 sup 11	2.06E+04	3.76E+03	5.5		4.52E+10	6.88E+10	1.52		
LV sup 1	7.32E+00	9.24E-01	7.9	2.82E+04	1.11E+07	2.44E+07	2.20	393	865
LV sup 2	4.91E+01	2.85E+00	17.2	1.03E+05	3.42E+07	1.64E+08	4.78	330	1581
LV sup 3	3.84E+02	5.54E+01	6.9	9.40E+05	6.65E+08	1.28E+09	1.92	708	1362
LV sup 4	8.14E+02	1.12E+02	7.2	1.72E+06	1.35E+09	2.71E+09	2.01	786	1580
LV sup 5	1.13E+03	1.21E+02	9.3	2.68E+06	1.45E+09	3.76E+09	2.59	541	1401
LV sup 6	1.66E+03	1.31E+02	12.7	2.39E+06	1.58E+09	5.54E+09	3.52	659	2319
LV sup 7	3.53E+03	3.78E+02	9.3	1.13E+07	4.54E+09	1.18E+10	2.60	402	1045

Table shows SG-PERT RT activity measured on the LightCycler® 480 and p24 antigen concentration in different productions of replication-competent HIV-1 virus supernatant (HIV-1, sup 1–11) and replication-incompetent HIV-1-based MISSION® lentiviral vectors (LV, sup 1–7). TU/mL: transducing units/mL (only determined for lentiviral vectors). The number of viral particles (#VP) was calculated from p24 values by assuming 12 viral particles per fg p24. For viral particle calculation from RT activity values, an activity of 300 pU per viral particle was assumed.

The MISSION® lentiviral particles used in this experiment express eGFP as a marker gene and therefore allow easy determination of TU/mL, by FACS measurement of the proportion of transduced cells. The number of TU/mL obtained after limiting dilution of the different lentiviral supernatants on Jurkat E6-1 cells strongly correlated with the RT activity levels in these samples (Figure 4B). When calculating the number of viral particles in the supernatant, assuming an RT activity of 300 pU per virion [9], [18], we found a ratio of 865 to 2,319 RT-containing viral particles per functional transducing unit. Viral particle numbers in the same range were obtained when calculations were based on p24 content, although the estimated particle concentration was slightly higher when based on RT activity (“RT based/p24 based” ratio of 1.5–3.8 for HIV-1 containing supernatant and 1.9–4.8 for lentiviral vector containing supernatant) (Table 1). This shows that as for p24, RT activity is representing both transducing and non-transducing viral particles.

For replication competent HIV-1 virus, relative levels of infectious units in the supernatants were assessed by infection of P4.R5 MAGI indicator cells. These CD4, CXCR4 and CCR5 expressing HeLa cells express bacterial β-galactosidase under control of the Tat-responsive HIV long terminal repeat and therefore express β-galactosidase upon productive HIV infection [33]. The levels of β-galactosidase activity induced by HIV-1 supernatant in these types of assays have been shown to correlate with infectious titers determined by end-point dilution (tissue culture infectious dose 50% or TCID50) [39]. When infecting the cells with a serial dilution of HIV-1 supernatant, the input viral concentration linearly correlated with the β-galactosidase activity measured in the cell culture 48 h after single-cycle infection. For high levels of viral input a saturation of β -galactosidase activity occurred at a level corresponding to an optical density value around 2 (Fig. 4C). To compare levels of infectious units present in different productions of HIV-1 NL4-3 viral supernatant, an appropriate dilution of each supernatant was used for single cycle infection. We found a high correlation between the levels of β-galactosidase activity and the RT activity, indicating a correlation between the number of infectious units and RT activity in the supernatant (Fig. 4D).

Furthermore, we evaluated the inter-run variation of p24 concentration measurement by ELISA and compared this to the SG-PERT inter-run variation determined above. For 7 different HIV-1 containing samples, from the same set that was used to determine SG-PERT reproducibility, p24 concentration was determined in at least 3 independent ELISA experiments. Due to the restricted linear range of p24 ELISA, careful consideration was given to appropriate dilution of the supernatants in each experiment. Coefficient of variation ranged from 16% to 51.4% and was on average higher than the inter-run variation of the SG-PERT assay (p-value = 0.0426 with Mann-Withney U test) (Figure 4E–F and compare to Figure 3C). Quantification of the sample with low viral titer and high inter-run variation of RT activity determination (sample 1 in Figure 4E), was only slightly more reproducible with p24 ELISA compared to SG-PERT (Figure 4E, compare to Figure 3C).

### SG-PERT Assay for Applied Biosystems qPCR Instruments

**Figure 5:**
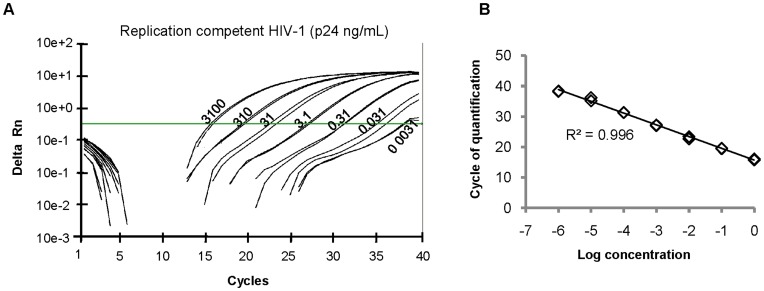
SG-PERT assay on ABI 7300 Real-Time PCR System. (A) Amplification curves of indicated amount of replication competent HIV-1 (NL4-3 strain) (ng p24/mL) obtained by SG-PERT on the ABI 7300 instrument. The horizontal line represents the threshold line used to calculate Cq values. (B) Correlation between input levels of replication-competent HIV-1 virus and Cq values obtained by SG-PERT on the ABI 7300 instrument. p24 antigen concentration in the undiluted samples (value “0” on x-axis) was 3,100 ng/mL.

For the characterization of the SG-PERT assay above we used the Roche SYBR Green I Master Mix for qPCR quantification on the LightCycler. However, other qPCR platforms, such as most of the Applied Biosystems qPCR instruments, require the presence of a passive reference dye in the master mix to normalize for non-PCR related fluorescence signal variations. To compare, we performed the assay on an ABI 7300 Real-Time PCR System using the ROX containing qPCR core kit for SYBR Green I from Eurogentec as reaction mix (see material and methods). Sensitivity and linear range of the assay were determined by using the same 10-fold serial dilution of HIV-1 supernatant as was used before on the LightCycler for this purpose. Similar to the results on the LightCycler, the SG-PERT assay could quantify RT activity in a sample with p24 antigen concentration as low as 0.0031 ng/mL and Ct values correlated linearly with the input virus dilution over six orders of magnitude (Figure 5A and 5B). When using samples with even higher RT activity, by using recombinant RT, we did notice a loss of linearity, probably by saturation of the assay (data not shown). However, such RT activity levels are far above the ones commonly obtained in supernatant of viral productions or medium of HIV infected cells. Nuclease-free water did not generate a detectable signal on the ABI 7300 instrument when used as input for SG-PERT (Figure 5A). Furthermore, melting curve analysis of the PCR products confirmed the absence of non-specific amplification in the assay (data not shown). Therefore, retroviral quantification by SG-PERT can be performed on ABI qPCR instruments with equal efficiency as on the LightCycler.

## Discussion

Assessment of retroviral titers is a requisite in quality assurance, virology and molecular biology research laboratories for quality control of cell derived preparations; quality control of viral productions; for standardization of experiments using different batches of virus preparations and for normalization of viral particles numbers when comparing different types of viruses. In this paper, we present the first one-step SYBR Green I qPCR-based PERT assay using commercial ready-to-use reaction mixes and show that quantification of RT activity in viral supernatant by this assay, can provide a robust and accurate alternative for other frequently applied methods of retroviral titer determination.

The SG-PERT assay presented here, uses MS2 RNA as a substrate for reverse transcription by retrovirus-associated RT molecules and subsequently quantifies MS2 cDNA by SYBR Green I-based qPCR, using commercially available reagents and ready-to-use reaction mixes. Since the introduction of a qPCR cDNA quantification step to conventional PERT, a number of assays applying this modification have been published [16], [17], [18], [19], [20], [21]. Most of these assays follow a two-step protocol, separating the reverse transcription and qPCR step [17], [18], [19], [20]. While such a separation offers the possibility to remove non reverse transcribed RNA templates after the RT step, thereby avoiding possible background signals of the Taq polymerase [20], it renders the assay more labor-intensive and time-consuming. For quantification of retroviral titers in research laboratories, easiness and speed of the assay is often more important than the elimination of minute background levels, that are often far below the signal generated by the retroviral supernatant. Based on assay durations reported in literature, the SG-PERT assay described here seems considerably faster compared to other published two-step and one-step qPCR-based PERT assays [16], [18], [20]. Furthermore, most available qPCR based PERT assay use cDNA-specific fluorogenic labeled probes (Taqman® chemistry) for signal generation [16], [17], [18], [19], [20], while SYBR Green I chemistry is more accessible and is not sensitive to possible DNAse activity present in the test samples, as has been reported for fluorogenic labeled probes in one-step qPCR based PERT assays [17]. Subsequently, the ability to perform the SG-PERT assay with commercial ready-to-use SYBR Green I qPCR reaction mixes largely facilitates its implementation in laboratories equipped for qPCR and eliminates possible compositional variation inherent to in house prepared qPCR mixes. Of note, we have confirmed that another commercially available SYBR Green PCR ready mix (the widely used Qiagen QuantiTect SYBR Green PCR Kit), is fully compatible with the linear quantification of HIV-1 RT activity and therefore most likely is an alternative to the Roche and Eurogentec mixes evaluated in this report.

During evaluation of the one-step SG-PERT assay, we found that obtained RT activity values correlated with input levels over six to seven orders of magnitude. Because of this extraordinary linear range, prior extensive serial dilution of the viral supernatant is not required for titer determination, thus potential introduction of variation is avoided. The sensitivity of the assay corresponded to a p24 equivalent of 3 pg/mL for HIV-1 supernatant and to ±50 or ±110 TU/mL for MoMLV-based and HIV-based retroviral vectors respectively. The original SG-PERT assay described by Pizzato et al. was reported to have a sensitivity of 10^2^–10^3^ pU recombinant HIV-1 RT per reaction [21]. Although similar input levels were still detected in the current modified assay on the LightCycler® 480, the obtained values were outside the linear range of the assay. The higher sensitivity observed by Pizzato et al. could be due to the use of a different qPCR instrument, the BMV RNA template-primer combination or the performance of home-made PCR reaction mix. Nevertheless, the sensitivity of the current assay is sufficient for most applications in virology research laboratories and similar to the one of most commercial p24 ELISA kits (eg. Innogenetics INNOTEST®: 10 pg/mL; Perkin-Elmer ALLIANCE® test: 12.5 pg/mL). The assay was furthermore able to accurately quantify RT activity in samples containing retroviruses of different origins (HIV-1 and MoMLV). This offers the advantage that viral (vector) preparations of different origins can all be evaluated within a single assay. In this regard, Ma et al. recently demonstrated that a standard curve of recombinant HIV-1 RT can be used to perform absolute quantification of other types of retroviruses by qPCR-based PERT [18], although the efficiency of different viral reverse transcriptase enzymes should be evaluated in our SG-PERT assay. However, for routine evaluation of a specific type of retrovirus, we recommend to determine absolute RT activity levels of a high-titer preparation of this virus by running a standard curve of the appropriate recombinant RT in parallel. For subsequent assays, a dilution series of this high-titer preparation can then be used as a standard curve.

Due to the accurate quantification capacity of qPCR, a low intra- and inter-run variation has been reported for different PERT assays [17], [18], [21]. However, variation is usually determined on the obtained Cq values and consequently underestimates the variation on the actual RT activity values. Therefore, we expressed variation of the SG-PERT assay as percentage of the actual RT values, which are calculated from the obtained Cq values by running a standard curve with known RT activity in parallel. We show an acceptable intra- and inter-run reproducibility of the assay, although, as expected, an increase in variation is observed in regions close to the detection limit of the assay. Inter-run variation of viral titer determination was on average lower when using the SG-PERT assay compared to p24 ELISA. A high inter-run variation for p24 antigen quantification by ELISA has been reported by others [5], [40], and might be due to extensive sample dilution or the multiple handling steps inherent to the assay, which are both sources of variance introduction.

Since the use of qPCR-based PERT assays as a retroviral titration method is still limited, one goal of this paper is to evaluate the informative value of retroviral titers based on RT activity levels. We show an excellent correlation of RT activity with both p24 antigen concentration and levels of transducing and infectious units.

For replication-incompetent lentiviral vectors, we calculated the number of viral particles from the obtained RT activity and found an average 1/1,450 ratio between transducing units and RT-containing particles. A functional/physical particle ratio in the same range has been reported for lentiviral vectors, when the latter was determined by quantification of viral RNA copies [5], [41], [42], [43] or p24 antigen concentration [44], [45]. However, these ratios are highly dependent on different characteristics of the both the vector and transduction process, such as the vector backbone and envelope protein, the transduction method and the cell line used to determine functional titers [5], [43]. Therefore, it is important to note that the correlation between RT activity and levels of transducing units was evaluated on lentiviral vector preparations produced with the same transfer plasmid, although the packaging plasmids and transfection methods used were different. We also calculated the ratio between the absolute number of infectious units and RT-containing viral particles for two productions of replication competent HIV-1 virus. The former was determined by analysis of the HSA marker gene expression, encoded by HIV-1 replication competent reporter viruses, in P4.R5 cells infected with a serial dilution of supernatant, the latter based on an RT activity of 300 pU per virion [9], [18]. We found ratios of 1/63,500 and 1/111,800 between infectious units and total viral particles (data not shown). In line with replication-incompetent lentiviral vectors, highly variable ratios of infectious/noninfectious units have been reported in literature for *in vitro* produced HIV-1 supernatant (ranging from 1/10^2^ to 1/10^7^) [46], [47], [48], [49]. These ratios are influenced by several factors, such as the type of virion producing cell, type and density of the target cell, the infection protocol and the HIV-1 strain [40], [48], [49], [50], [51]. If a precise estimation of the transducing or infectious capacity is necessary, it is therefore recommended to establish titers of the particular vector or HIV virus with a functional titration method immediately on the cell line of interest [5], [52]. However, if fixed transduction or infection levels are not necessary, a more rapid estimation of physical particle content, with for instance the SG-PERT assay, is often sufficient to assess quality control of the viral production or to normalize the number of viral particles before transduction or infection. It should be noted that the actual number of RT-containing viral particles might be different from the ones estimated in Table 1, since the RT activity per virion might be dependent on both the origin of the HIV RT packed in the viral particles as well as the source of the recombinant RT used for quantification in the SG-PERT assay. In addition, we cannot exclude that some of the RT activity is not bound to a viral particle. Nevertheless, these results indicate that, similar to the p24 antigen concentration, the virion associated RT activity most likely provides an estimate of the physical particle content in lentiviral supernatant, which strongly correlates with the functional particle content for both replication-incompetent lentiviral vectors and replication-competent HIV-1 virus.

Correlation between RT activity and infectious units of replication competent HIV-1 virus has been investigated by others in the past [39], [40], [53], [54]. Although one study found RT activity to be a poor predictor of virion infectivity [54], most of them observed a correlation between infectivity and the RT activity measured in the supernatant by conventional non-PERT RT assays [39], [40], [53]. It has to be noted that these studies were done on primary HIV isolates and the presence of correlation appeared to be dependent on whether or not viruses were grouped according to coreceptor usage or subtype [39], [40]. Since our study evaluated different productions of the same HIV-1 strain (NL4-3), a correlation between RT activity and relative levels of infectious units is in agreement with these studies and indicates that the SG-PERT assay is a fast and worthy alternative for non-PERT RT assays in these types of studies. In contrast to RT activity, p24 antigen levels show a poor correlation with levels of infectious HIV units in most studies [39], [40], [53], [54]. In this regard, it has been reported that RT activity might present an intermediate of physical and functional particle concentration. This was based on the observation that the levels of RT activity and infectious units in HIV-1 infected cell cultures over time show a rapid decrease after reaching peak levels, while p24 antigen levels further accumulate and subsequently reach a plateau phase. These authors therefore assume a short half life of RT activity, while p24 proteins are still detected upon decay of infectious particles [39], [40]. In the present study, p24-RT activity correlation was evaluated in supernatant collected from transfected 293T or 293TN cells at 2 or 3 days post transfection. Since the levels of transducing units in these preparations also correlated with p24 levels (data not shown), viral particle decay is probably still limited at this time-point [5]. However, when monitoring lentiviral replication levels over prolonged periods of time, assessment of RT activity levels might provide a more accurate result compared to p24 antigen levels.

Implementation of the SG-PERT assay in our laboratory was found to be easy. A lab equipped for qPCR assays only needs to acquire the MS2 RNA template, MS2 specific primers, and a small amount of the recombinant RT of interest. The Triton X-100 based lysis buffer can be prepared from routine chemical ingredients and a standard curve can be established from any high-titer retroviral preparation of choice. We calculated that the current reagents cost of the SG-PERT assay using the LightCycler® 480 and associated reaction mix is about 10 times lower per retroviral quantification compared to determination of p24 antigen levels with a commercial ELISA kit. Moreover, since the limited linear range of quantification of the ELISA assay often requires the measurement of 3 different dilutions per sample, cost and labor increase even more. Furthermore, while titer quantification of 30 samples by ELISA can take up to six hours of hands-on labor time, the SG-PERT assay requires less than 2 hours hands-on time and is amenable to automation and further reduction in reaction volumes.

In summary, this paper shows that the SG-PERT assay with commercially available MS2 RNA and qPCR reaction mixes is a robust and accurate method for retroviral quantification. Titer determination by this assay correlates well with those of other frequently applied methods. Combined with its low cost, fast procedure and easy implementation, it is an attractive alternative for use in virology and molecular biology research.
